# Offshore Online Measurements of Total Suspended Solids Using Microscopy Analyzers

**DOI:** 10.3390/s21093192

**Published:** 2021-05-04

**Authors:** Dennis Severin Hansen, Stefan Jespersen, Mads Valentin Bram, Zhenyu Yang

**Affiliations:** Department of Energy Technology, Aalborg University, Niels Bohrs Vej 8, 6700 Esbjerg, Denmark; dsh@et.aau.dk (D.S.H.); sje@et.aau.dk (S.J.); mvb@et.aau.dk (M.V.B.)

**Keywords:** oil and gas industry, produced water, microscopy analyzer, oil-in-water concentration, real-time measurements, calibration procedure

## Abstract

Accurate online water quality measurements have gained attention during the last decades in the oil and gas industry for improving operational performance and protecting the surrounding environment. One potential solution to extend the reservoirs’ economic life and put less strain on the environment is by re-injecting the produced water, but the injected water quality must be high and consistent to prevent injectivity reduction. This paper evaluates two different online microscopy analyzers that utilize a high-resolution video camera for capturing images of the particles passing their view cell. The calibration procedure for both online microscopy analyzers has been thoroughly validated for steady-state and real-time measurements. The real-time measurements were achieved by post-processing the data captured by the microscopes and applying a trailing moving average window. The performance of measuring the oil-in-water concentration was compared with an online fluorescence-based monitor. The paper addresses the statistical considerations when defining the level of accuracy of the predicted particle size distribution within a defined confidence interval. Both microscopes showed promising results for measuring known particle sizes and oil-in-water concentrations, both in steady-state and real-time.

## 1. Introduction

Even as a general global goal is to consume energy from renewable energy sources, oil and gas are needed in the transition [1]. Oil production is expected to increase during the next three decades globally, which entails the oil and gas industry to significantly impact the world’s energy consumption in the coming decades [2]. The general trend towards more sustainable energy production also affects the offshore oil and gas industry as discharge legislation becomes stricter [3,4]. With stricter policies, produced water re-injection (PWRI) has gained growing attention to extend the reservoirs’ economic life and decrease produced water (PW) discharge to minimize environmental impacts [5]. However, the re-injected PW and the injected seawater must have a continually high quality to prevent formation damage and unpredicted injectivity reduction [6,7,8,9,10]. Currently, only ∼14.5% of the PW in the Danish sector of the North Sea is re-injected, which is a >50% reduction since 2009 due to reservoir challenges according to the Danish Environmental Protection Agency [3,11]. To increase the PWRI percentage, efficient management is essential to maintain a high PW and seawater quality, which involves proper treatment and accurate monitoring. Monitoring the PW also support protecting the receiving environment when discharging. Accurate information on the amount of oil and particles, sizes, and classification of particles in the injection water (IW) can essentially be used for decision support, reporting, or even advanced control to achieve better operation in the treatment process [6,12]. Therefore, the importance and awareness of accurate online measurements of oil and suspended particles have gained increasing attention within the oil and gas industry [5,6].

The water quality in offshore injection water treatment (IWT) processes is usually assessed in terms of particles’ plugging tendency, also addressed as suspended solids or total suspended solids (TSS) [8,13]. However, the IW quality should be economically viable to achieve and counterbalanced against other solutions, e.g., well-stimulation [12,13]. Currently, offshore IWT processes rely on off-line measurements of TSS concentration following the ISO 11923; thus, in case of a decrease in IW quality, the reaction time is long due to the use of onshore laboratory measurements [14].

Even if at-line execution significantly reduces the reaction time of off-line TSS concentration measurements, it is difficult for operators to investigate where and what is the root cause of the decreased quality when there is no available information related to TSS. Especially as significant water quality decline often occur downstream [8]. Online TSS and oil-in-water (OiW) concentration measurements have not become standardized in the oil and gas industry, despite the long history of measuring particle sizes online [14]. The measurement technique is often not the main reason for the error source in the observed results [15]. Different studies emphasize that the main source of errors often are due to improper installment and calibration [15,16,17].

According to Latif [18] only a few research studies of continuous measurement techniques for online concentration measurements have been published. Latif [18] and Fjords Processing [19] have done a comparison study on different techniques for measuring OiW concentration. However, their focus is on how different variations of parameters such as mixtures, pressures, chemicals, and temperatures can affect the measurements and not on the uncertainty related to the petroleum engineers’ ability to determine an acceptable calibration. Although PWRI is a solution for extending the economic life of oil production, it is beneficial to measure particle sizes to increase IWT processes’ performance. Several different methods for measuring particle sizes exist, each based on several design options from different manufacturers. Besides microscopy, which is based on direct observation, all other techniques are challenged by their property assumptions that only the equivalent diameter of a sphere is measured [12]. The existing morphologies are not taken into account, which complicates the ability to classify particles [12]. Another advantage of microscopy is the manual discrimination of particles captured to evaluate the results. This paper aims to examine two different online microscopy analyzers: Jorin ViPA and Canty InFlow.

Both microscopes are based on the same technique of utilizing a high-resolution video camera to capture images of the particles passing the view cell. Both monitors are based on bright field illumination techniques, presenting a dark image of particles passing with a bright background. The pixel scale of Jorin ViPA is fixed by the manufacturer, and the Canty InFlow is adjusted to a specific pixel scale. A fluorescence-based monitor (Turner TD-4100XDC) is used as a benchmark to evaluate the two online microscopes’ performance to measure OiW concentrations. The fluorescence-based monitor is selected based on a previous work by Hansen et al. [6]. This paper addresses the problems occurring when petroleum engineers must subjectively decide what particles are considered in focus. Furthermore, the calibration procedure will be validated on known solid particle sizes and their ability to measure different OiW concentrations accurately and in real-time. Especially to determine the accurate volumetric concentration in each image is not trivial as the depth of field (DoF) would affect the estimate of particle concentration.

## 2. Materials and Methods

**Figure 1:**
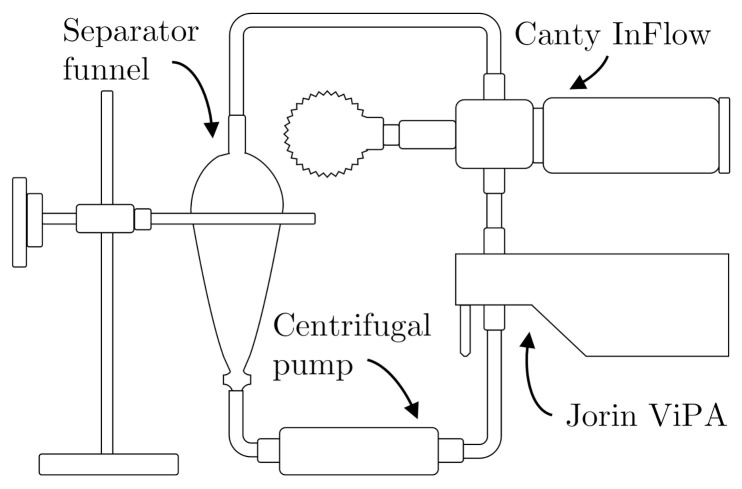
A recirculating test setup for measuring known particle sizes to calibrate both online microscopes.

**Figure 2:**
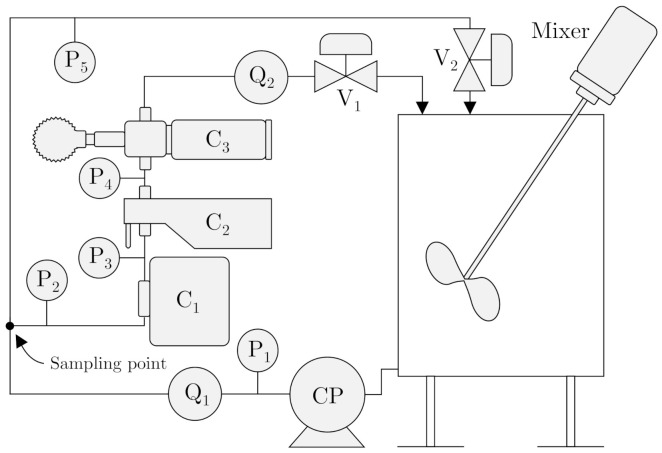
Skid mounted testing platform for validating quality monitors’ performance on a sidestream installation during different flow regimes.

Two setups were constructed to execute the experiments presented in this paper. The two systems are shown in Figure 1 and Figure 2. The setup shown in Figure 1 is used to calibrate both online microscopes using different known polystyrene particle sizes produced by BS-Partikel [20].

The three different polystyrene particle sizes follow a statistically Gaussian distribution, and the measured means and standard deviations presented in parentheses, are given by the manufacturer:

- Nominal diameter of 10µm: $x_{3}=9.8\left(0.3\right)$µm
- Nominal diameter of 20µm: $x_{1}=20.1\left(0.4\right)$µm
- Nominal diameter of 40µm: $x_{2}=40.3\left(0.9\right)$µm

Figure 2 illustrates a skid-mounted testing platform for validating the two online microscopes’ performance. The platform is equipped with a centrifugal pump (CP), pressure transmitters (P_x_), flowmeters (Q_x_), and pneumatic control valves (V_x_). The system is configurable to direct the liquid through the sidestream and the mainstream by manipulating the control valves.

By taking advantage of on-line sampling, both microscopes: Jorin Visual Process Analysis (ViPA) B HiFlo (C_2_) and Canty InFlow VD4912-960 (C_3_) are installed on a sidestream, making them applicable in most installations regardless of the flow velocity and the pipeline’s dimensions. Although, on-line measurement complicates the sampling procedure as a maldistribution between the run and the branch can occur.

Both microscopes mainly consist of a camera, light source, flow cell, optical lens, and a computer. For C_2_ a progressive CCD camera (Sony ICX445) with 1292×964 pixels captures images at maximum frame rate at full resolution of 31 frames per second (fps) with sensor pixel size of 3.75×3.75 µm. A Comar 10 OA 25 lens is used to output a field of view of 484.50×361.50 µm with a magnification of 10, a numerical aperture of 0.25, and a focal length of 16.9 mm. The manufacturer of C_3_ retains most of the equipment specifications confidential, however some general specifications of both microscopes are listed in Table 1.

A fluorescence-based monitor (C_1_) that is sensitive to the aromatic content is installed to measure the OiW concentration. C_1_ is installed as a benchmark to facilitate the investigation of measuring OiW concentrations by C_2_ and C_3_. The accuracy of C_1_ has been extensively studied by Hansen et al. [6], and will not be evaluated in this paper.

**Figure 3:**
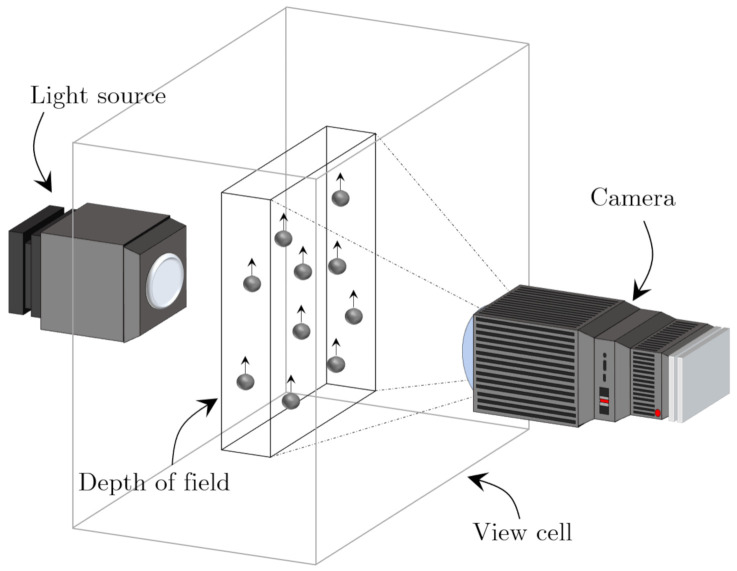
The depth of field of the measured volume within the view cell.

The difficulty of online microscopy analysis is related to the narrow focus area, where only a fraction of the entire flow is directed into the sidestream and through the view cell. A further complication occurs as only a narrow DoF of the passing flow is measured by the microscope; thus, not all TSS that passes the view cell will be observed and are therefore excluded from being measured as shown in Figure 3.

To achieve statistical results, it is necessary to sample a sufficient amount of particles that is constricted by the small DoF. The quantity of captured particles strongly relates to the accuracy of the predicted particle size distribution (PSD). The accuracy of the PSD can be determined within a region of relative error, δ, from a defined confidence level with its represented z-score value, *u*. The number of sufficient particles, $n^{*}$, that are required is based on δ and sample standard deviation, *s*, has been proposed by Masuda and Iinoya [21]. The approach is based upon the assumption that the PSD follows a log-normal distribution.

(1) $$\log\left(n_{*}\right)=-2\log\left(\delta \right)+\log\left(\omega \right),$$

where

(2) $$\omega =u^{2}\alpha^{2}s^{2}\left(2c^{2}s^{2}+1\right).$$

α is the non-zero exponential constant that defines the particle distribution, for log-normal distribution α=2 [22]. *c* can be calculated as

(3) $$c=\beta +\frac{\alpha }{2},$$

where β is a basis number, for the count basis β=0. The number of particles required within a certain range of error changes depending on how the data is scattered. Thus, the number of particles increases as the deviation of the data increases. Simultaneously, the number of particles increases proportionately to the error of magnitude required. On the contrary, the relative error can be estimated based on the number of particles observed.

### Experimental Design

This subsection describes the design and objective of all executed experiments on both setups from Figure 1 and Figure 2. The two microscopes were calibrated according to a known particle size from BS-Partikel, followed by a performance validation on known sizes, larger and smaller than the known particle size used for calibration. The two microscopes were installed in series together with the fluorescence-based monitor to observe their performance of measuring OiW concentrations. The following experiments are executed on the setup presented in Figure 1:

Experiment I: calibration of both microscopes, by addition of known particle sizes with a mean and standard deviation: $x_{1}=20.1\left(0.4\right)$ µm.
Experiment II: validation test of the calibration procedure executed in Experiment I, by addition of a known particle size larger and smaller than $x_{1}$:$x_{2}=40.3\left(0.9\right)$ µm and $x_{3}=9.8\left(0.3\right)$ µm.

The pump speed was fixed at the same value in both Experiment I and II, which generated a flow rate of ∼2 L/min. Most of the presented data in Experiment I are based on data from C_2_, although the exact same procedure was executed on C_3_. Both microscopes have three calibration parameters:

- Threshold value (THV): an 8-bit integer, resulting in a greyscale image with pixel values in the range of 0–255, from black to white for both microscopes.
- Edge strength/focus rejection value (ESV/FRV): an edge detection method, running a convolution kernel for estimating the gradient at each pixel on the image. The most common method is Sobel, which has been used on C_3_. Other methods are also available when using C_3_. C_2_’s edge detection method is confidential. The ESVs of C_2_ are in a range of 0–10, where ESV =10 only includes particles with a large gradient. C_3_’s FRV ranges from 0–1000, although FRV >45 did not include any particles in the calibration analysis.
- Depth of field (DoF): defines the depth of the captured images. DoF normally defines the distance between the closest and farthest particle in the image that appears acceptably sharp. DoF is only necessary to adjust if the user is interested in the sample’s concentration as the DoF influence to field of view volume. To determine the DoF for these microscopes, the DoF is adjusted to present the known concentration based on the accepted measured particles fulfilling THV and ESV/FRV. The DoF is then estimated using
  (4) $$C=\frac{\sum_{j=1}^{k}\sum_{i=1}^{n}\frac{1}{6}\pi d_{A_{i,j}}^{3}}{A_{FOV}\bar{z}k},$$
  where *C* is the known concentration, *k* is the number of images, *n* is the number of particles in each *j*th image, $d_{A_{i,j}}$ is the equivalent area diameter for each *i*th particle in each *j*-th image, $A_{FOV}$ is the field of view, and $\bar{z}$ is the average DoF. By adjusting $\bar{z}$ in (4) of the captured images within a defined time interval, it is possible to adjust the measured concentration from the microscopes to match the known concentration.

Only the first two calibration parameters were analyzed in Experiment I and validated in Experiment II by the addition of the particle sizes: $x_{2}$ and $x_{3}$. The DoF value was tuned in Experiment III. The following experiment is executed on the setup presented in Figure 2:

Experiment III: a performance evaluation of the microscopes, to measure OiW concentrations in steady-state and real-time measurements, benchmarked according to the known OiW concentration and the measurements obtained from C_1_. Six nominal OiW concentrations were investigated (55,100,150,200,250,400)ppm. The supply tank was filled with 163.59 L of 1 µm filtered tap water. The solution of oil added are listed in Table 2 for each OiW concentration.

CP was kept at 100% pump speed to ensure that the droplet size distribution does not change over time due to the high shear occurring in CP. V_2_ was kept at a fixed opening degree 37.5%, and the flow rate through the sidestream was kept constant by manipulating V_1_.

## 3. Results

The results are divided into three sections based on Experiment I, II, and III.

Experiment I: 

A 2 h recirculation experiment with particle size $x_{1}$ dispersed in 1 µm filtered tap water, was executed with both microscopes and used as calibration data.

Firstly, the THVs were adjusted so that the known particle size $x_{1}$ can statistically be represented by the microscopes. A high ESV was selected only to determine the THVs on captured particles in focus. Determining the THV before the ESV is essential as the edge detection method depends on the THV.

**Figure 4:**
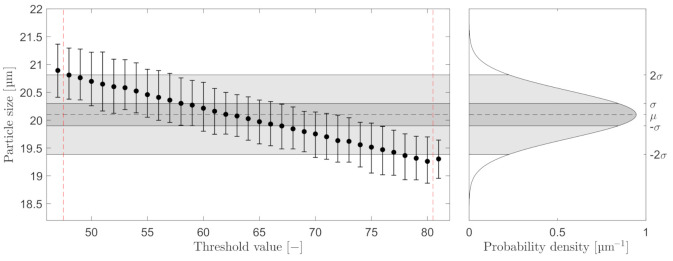
Selected particles, all with maximum ESV on C_2_. The error bars represent the mean, minimum, and maximum particle sizes at different THVs. The Gaussian distribution is calculated based on information from BS-Partikel: µ = 20.1 µm and = 0.4 µm.

Furthermore, for comparison of both microscopes’ performance, particle sizes were based on equivalent area diameter during post-processing of the data. Note that choosing another method of determining the particle diameter will most likely shift the distribution of measured sizes. The measurement in Figure 4 presents the mean results of the first 100 images with particles captured at maximum ESV. The THV was then toggled in a range of 47–81, where at THV ≤47 and THV ≥81, particles no longer fulfill the ESV’s criteria and are no longer counted. The Gaussian distribution in Figure 4 represent the information analyzed by BS-Partikel: μ=20.1 µm and σ=0.4 µm.

**Figure 5:**
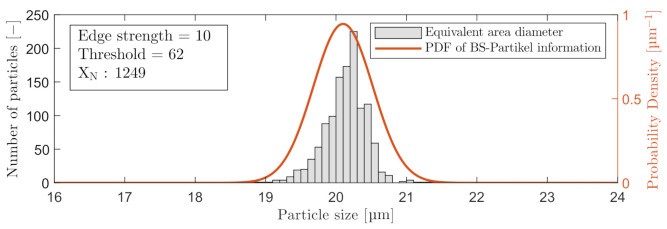
Included particles with a THV = 62 and ESV = 10, measured by C_2_.

Based on the results in Figure 4, a THV =62 for C_2_ was selected. For C_3_, a THV =182 was selected. The analysis results with a THV =62 and maximum ESV =10 are shown in Figure 5. A probability density function (PDF) is included on the graphs in Figure 5, to visualize the performance of estimating the particle sizes.

**Figure 6:**
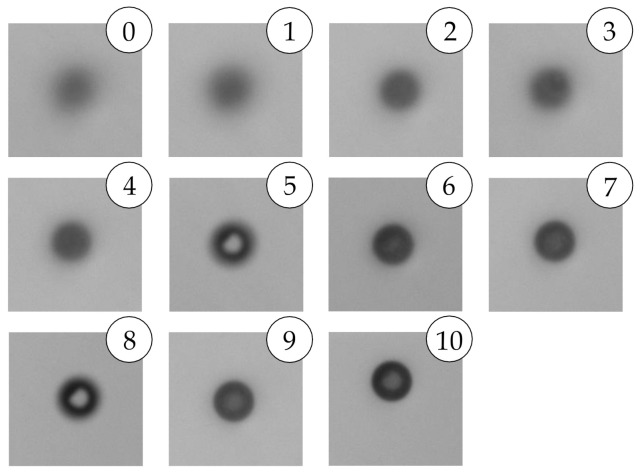
Eleven different particles were captured by C_2_ with a THV = 62, each with different acceptable ESV between 0–10. Associated results based on ESV are shown in Table 3.

Selecting the ESV is more subjective as it is a perception of what appears to be acceptably sharp. Figure 6 presents eleven different particles captured by C_2_ with a THV =62, each with different acceptable ESV between 0–10. Associated information of each particle, presented in Figure 6, is shown in Table 3.

**Figure 7:**
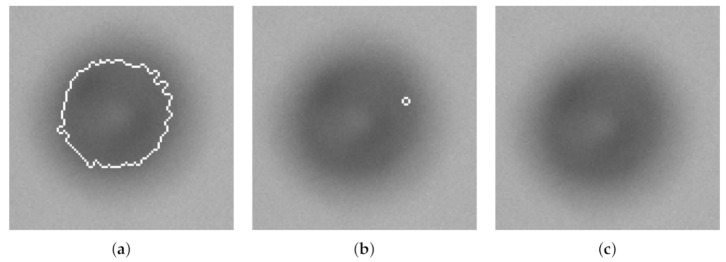
Three images of the same particle with a THV = 62, but with different ESVs: (**a**) ESV ≤ 1; (**b**) ESV = 2; (**c**) ESV ≥ 3. Associated results are shown in Table 4.

Furthermore, Figure 7 and Table 4 show an artifact of selecting a weak ESV. Even though an ESV of one outputs a measured size within 1σ from the μ of $x_{1}$, selecting an ESV =2, the gradient of the particle is no longer accepted and is considered out-of-focus. Although a smaller, but stronger gradient within the particle, fulfills the requirement and is included as a particle.

**Figure 8:**
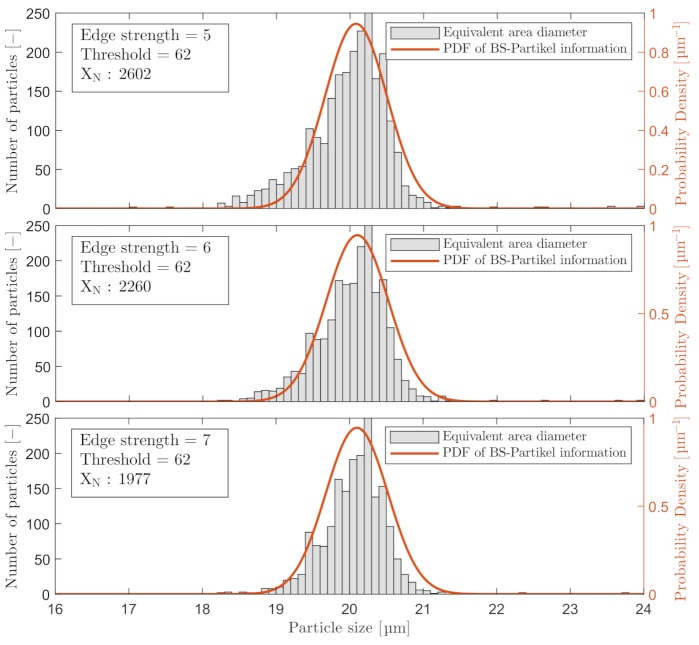
Included particles with a THV = 62 and three different ESVs: 5, 6, and 7, respectively, measured by C_2_.

Selecting a too high ESV will reduce the counted number of accepted particles in the analysis, or even worse, not represent other types of particles in the process that has a weaker gradient by nature. A further validation of selecting a proper ESV or FRV was executed by analyzing the corresponding results of the first three particles in Table 3 that are within 2σ from μ of $x_{1}$ (ESV: 5, 6, and 7). The results are shown in Figure 8. The same evaluation procedure was executed on C_3_.

**Figure 9:**
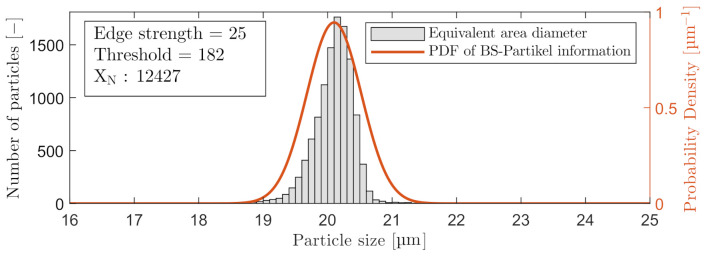
Included particles with a THV = 182 and a FRV = 25, measured by C_3_.

Increasing the ESV from 5 to 7 counted 24% fewer particles in Experiment I. Another phenomenon occurs at ESV =5, as the size histogram is skewed left due to the acceptable edge is found closer to the center of the particles as the peripheries are less in focus. Selecting between an ESV =6 or ESV =7 is a tradeoff between obtaining less countable particles and reducing the phenomena of underestimating the size of particles that are less in focus. ESV =6 was chosen for C_2_, and FRV =25 was chosen for C_3_. The calibration results of C_3_ are shown in Figure 9. The number of particles captured by C_3_ was ∼5 times larger than the amount captured by C_2_. This is primarily a result of higher image resolution and larger pixel size than C_2_ as shown in Table 1.

**Figure 10:**
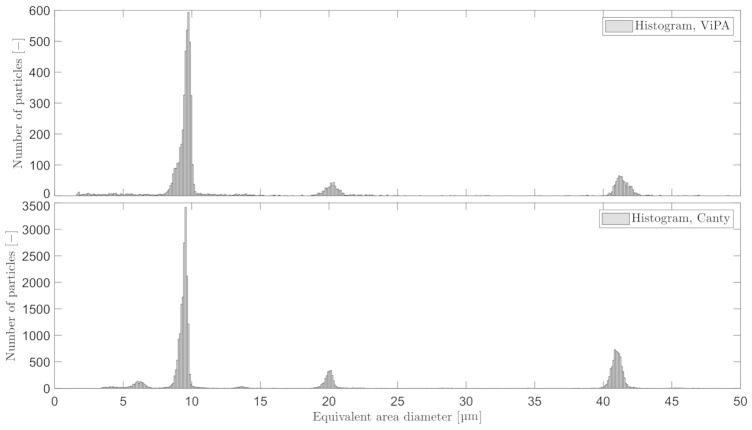
Three known particle sizes were measured simultaneously by C_2_ and C_3_. The mean and sample standard deviation are listed in Table 5.

Experiment II: 

A 2 h validation test was executed by adding two additional particle sizes: $x_{2}$ and $x_{3}$, along with $x_{1}$. The raw data from C_2_ and C_3_, without any classification, are shown in Figure 10.

For each known particle size, 1 ml aqueous surfactant solution containing the polystyrene particles was added. Both microscopes observed a high number of $x_{3}$ in the validation test, a result of containing more particles per volume than the others. By truncating the sample for each known particle size with a fixed range of ±4 µm from the known μ of $x_{1}$, $x_{2}$, and $x_{3}$, the statistical information can be obtained as shown in Table 5.

Experiment III: 

A 9.5 h experiment was carried out with the addition of oil after at least 30 min between each OiW concentration of observation.

**Figure 11:**
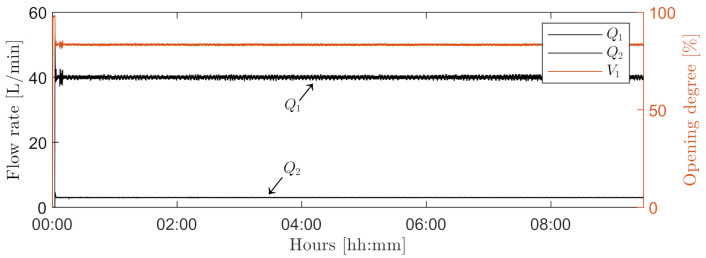
Flow rates from Q_1_, Q_2_, and the opening the degree of V_1_ to maintain a constant flow rate through the sidestream of the entire ∼9.5 h experiment.

**Figure 12:**
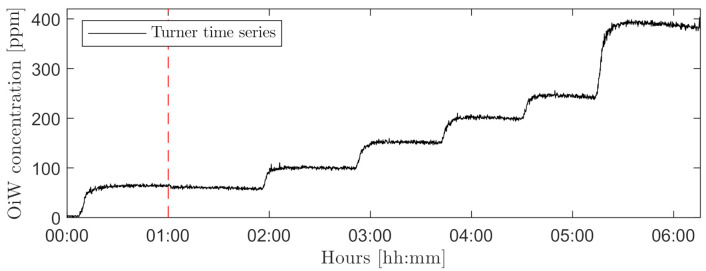
Time series of measured OiW concentration measured by C_1_ executed on the setup shown in Figure 2. The vertical line marks the truncated time series of ∼3.5 h.

Figure 11 shows flow rates of the mainstream (Q_1_) and sidestream (Q_2_), and the opening degree of V_1_. The sidestream was sufficiently kept constant at 3 L/min during the entire experiment by manipulation of V_1_. Figure 12 shows the time series of measured OiW concentration from C_1_. The vertical line marks a truncated time series of ∼3.5 h that was left out. The DoF value in C_2_ and C_3_ was at that period adjusted to match a OiW concentration of 55 ppm during the first 30 min after the injection of oil into the process by use of (4) and to classify oil droplets. The DoF value in C_2_ and C_3_ was estimated to be 43.14 µm and 94.29 µm, respectively.

**Figure 13:**
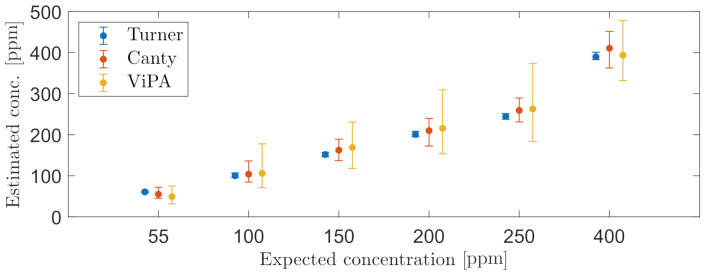
Error bars representing the mean, minimum, and maximum OiW concentrations based on the average concentration obtained each minute from C_2_ and C_3_, together with error bars based on measurement every 10 s from C_1_.

During the last known OiW concentration of 400ppm, the mixed concentration can no longer be maintained in the setup according to the measurement of C_1_. Figure 13 presents an error bar of each OiW concentration measured in a duration of 30 min for each online monitor. Each error bar represents the mean, minimum, and maximum OiW concentration measured, where C_1_ was selected to have a sampling frequency of 0.1 Hz, and C_2_ and C_3_ outputting an averaged concentration measurement every minute. Table 6 shows the associated results related to Figure 13.

**Figure 14:**
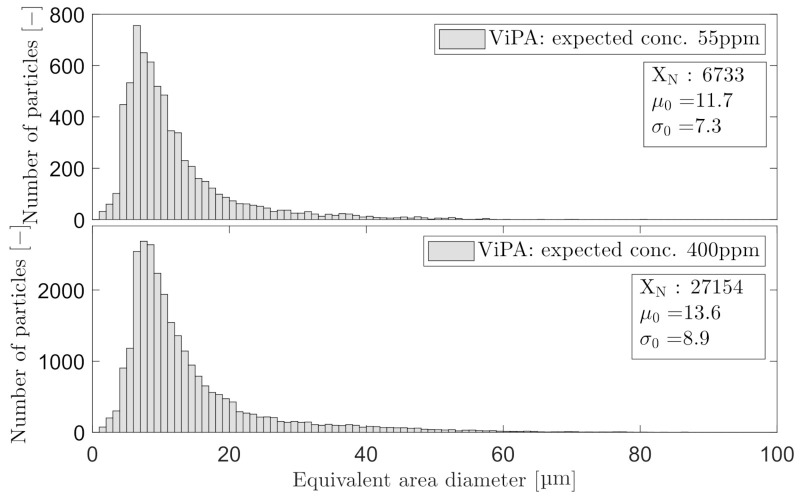
Droplet size distribution obtained with C_2_ based on the measured OiW concentrations at the known concentration of 55 ppm and 400 ppm, respectively.

**Figure 15:**
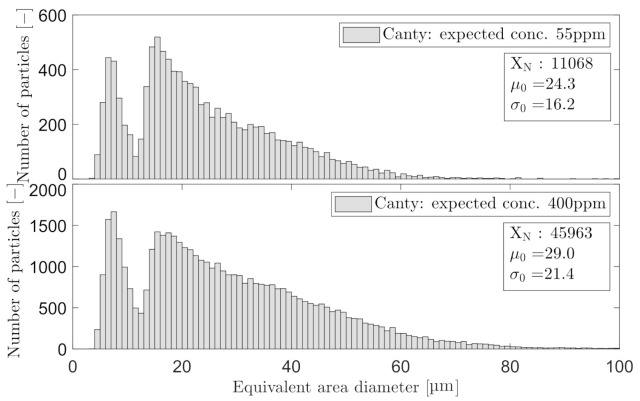
Droplet size distribution obtained with C_3_ based on the measured OiW concentrations at the known concentration of 55 ppm and 400 ppm, respectively.

Figure 14 and Figure 15 present the PSD histogram of the measured OiW concentrations at 55 ppm and 400 ppm for each microscope. Furthermore, the Figure 14 and Figure 15 presents the mean of the log-normal distribution, $\mu_{0}$, the standard deviation of the log-normal distribution, $\sigma_{0}$, and the number of counted particles, $X_{N}$.

Both OiW concentrations follow a log-normal size distribution measured by both microscopes, with only small changes in the distribution with respect to the concentration. Following the proposed calculation by Masuda and Iinoya [21] in (1)–(3), for obtaining a sufficient statistical representation within 95% confidence level, δ of the PSD can be calculated as shown in Table 7.

**Figure 16:**
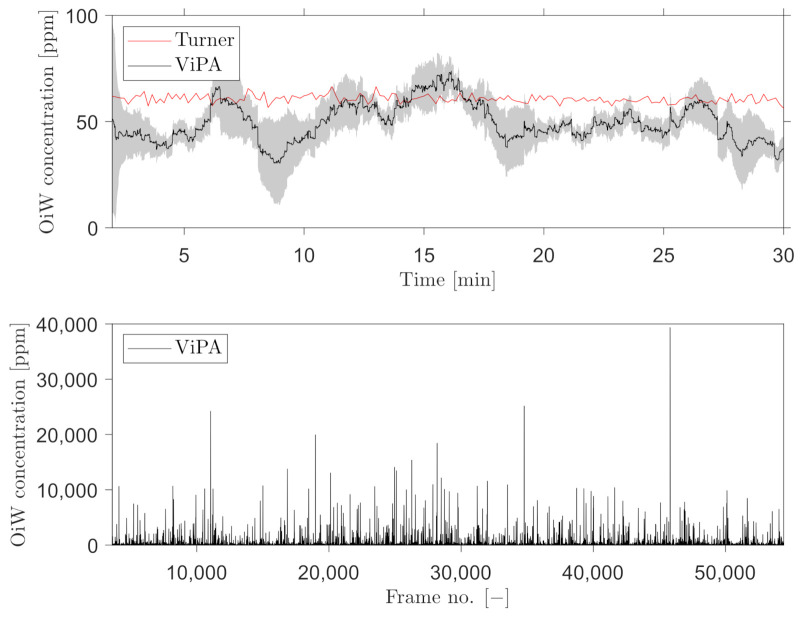
Real-time OiW concentration measurements calculated using a trailing moving average window of 1 min of the post-processed oil volume ratio in each image captured by C_2_. The top graph shows the real-time measurement measured by C_1_ and C_2_. A 95% confidence interval of the averaging window is shadowed behind the signal. Bottom graph shows the volume concentration of each frame captured.

**Figure 17:**
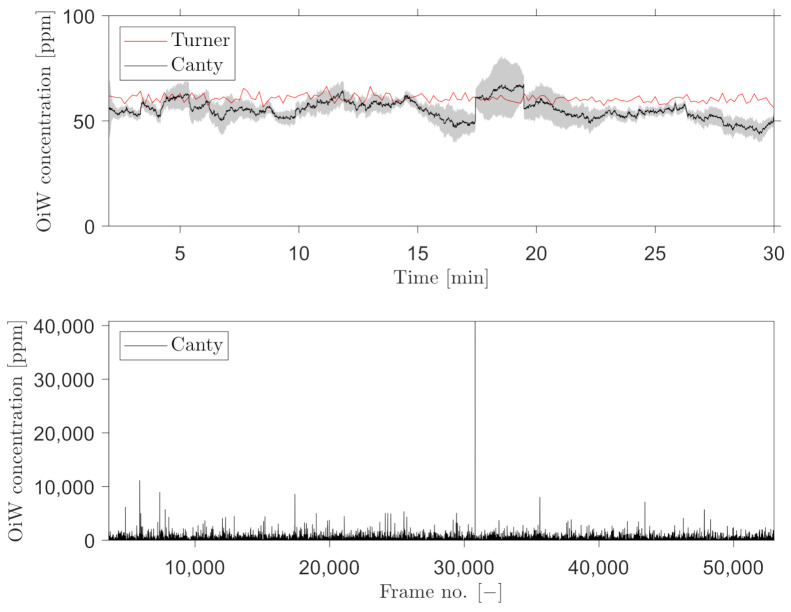
Real-time OiW concentration measurements calculated using a trailing moving average window of 1 min of the post-processed oil volume ratio in each image captured by C_3_. Top graph shows the real-time measurement measured by C_1_ and C_3_. A 95% confidence interval of the averaging window is shadowed behind the signal. Bottom graph shows the volume concentration of each frame captured.

A trailing moving average window of 1 min was selected, for measuring the OiW concentration real-time by C_2_ and C_3_. Figure 16 and Figure 17 show the results of real-time measurements at the known OiW concentration of 55 ppm from C_2_ and C_3_, respectively, together with real-time measurements from C_1_. The calculated OiW concentration, based on post-processed equivalent volume measurements, are presented for each captured image.

**Figure 18:**
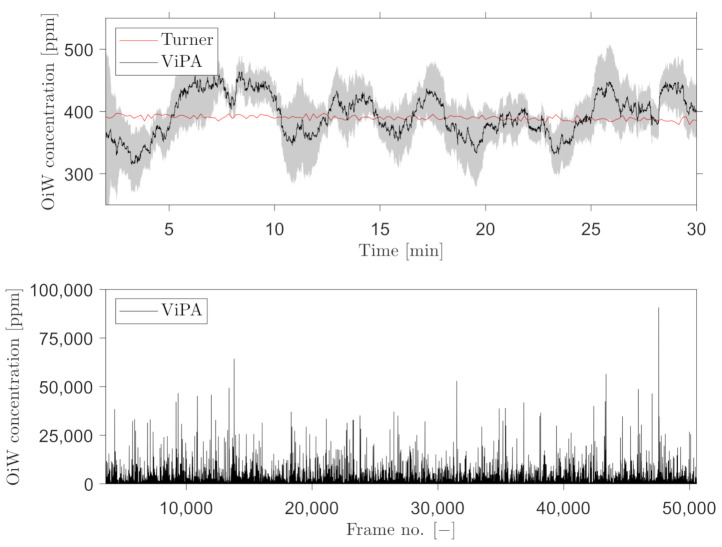
Same procedure as in Figure 16 for real-time OiW concentration measurement based on captured images from C_2_ at another measurement range of ∼400 ppm.

**Figure 19:**
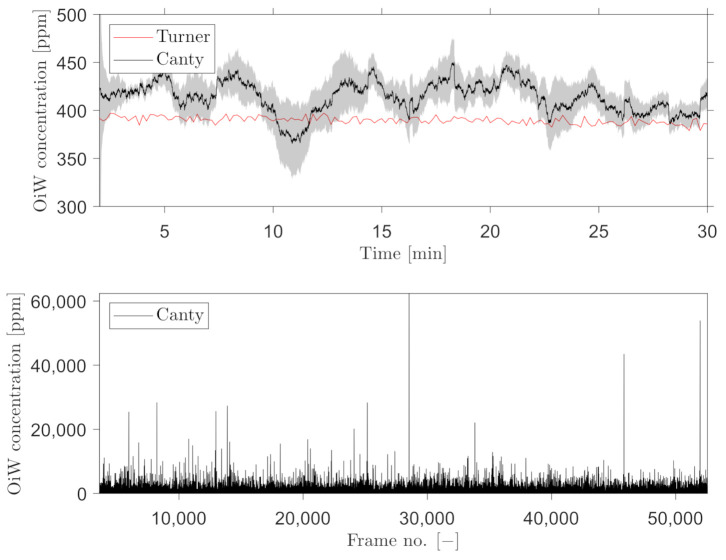
Same procedure as in Figure 17 for real-time OiW concentration measurement based on captured images from C_3_ at another measurement range of ∼400 ppm.

The results presented in Figure 16 and Figure 17 have also been executed at the known OiW concentration of 400ppm and presented in Figure 18 and Figure 19.

## 4. Discussion

The process of calibrating the two online microscopes is highly based on the perception of what is considered in focus. To ensure consistent results when calibrating the three main calibration parameters: THV, ESV/FRV, and DoF, the calibrating procedure must be well documented to reduce the difference between each calibration execution. Figure 4 and Figure 8 presented one solution for selecting the THV and ESV/FRV that outputs an estimation that matches the Gaussian distribution of the known particle sizes based on the information from the manufacturer of the produced particles. The skewness observed in Figure 8 when calibrating the ESV could result from selecting a slightly too low THV; thus, a lower ESV may have yielded better results. Although, based on the results shown in Figure 10 the calibration procedure presented in Experiment I revealed only a small deviation from the known PSDs. To further improve online microscopy analyzers, auto-calibration or human-computer interaction (HCI) of the calibration procedure can lower the subjective perception of what is acceptably sharp.

The steady-state values of measuring OiW concentration in Figure 13 showed promising results in relation to the known OiW concentrations. The measurement of C_1_ confirmed that C_2_ and C_3_ are able to measure the mean OiW concentrations by estimating the DoF using (4). The DoF for C_3_ was close to the theoretical estimation of DoF from the manufacturer seen in Table 1. The DoF for C_2_ was ∼63% lower than the theoretical estimation of DoF given by the manufacturer.

Moreover, C_1_ also confirmed that the setup was able to keep a well-mixed OiW concentration, apart from high OiW concentrations where a declining tendency of measured concentration was observed. The observed declining tendency may occur due to accumulation within the setup (dead volumes), natural separation in the supply tank, or pipelines and equipment becoming oil-wet.

**Figure 20:**
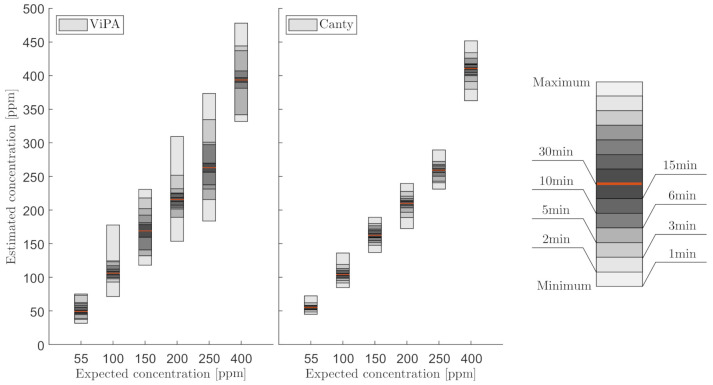
Error bars representing the minimum and maximum OiW concentrations based on different time durations of averaging the OiW concentrations obtained by C_2_ and C_3_, respectively. The right error bar illustration shows different time durations according to the greyscale value. The red indicator shows the grand mean of the OiW concentrations obtained within the execution time of 30 min.

Regardless of these sources of error, both microscopes were able to measure the mean OiW concentration of 30 min in close relation to the known concentration. A high deviation was observed when outputting OiW concentration every minute in steady-state, especially considering the steady concentration measured by C_1_ as shown in Figure 12. Figure 20 shows the results of extending the averaging time duration of both microscopes in steady-state. The results in Figure 20, show that increasing the averaging time to 3 min reduces the fluctuation of measured OiW concentration greatly. Meanwhile, the negative impact is that the time resolution of dynamics will be reduced accordingly.

**Figure 21:**
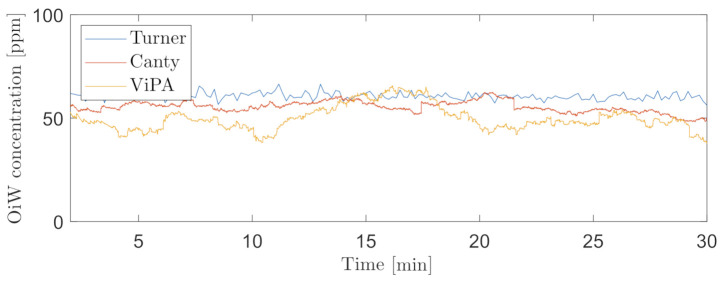
Real-time OiW concentration measurements calculated extending the trailing moving average window to 3 min for both C_2_ and C_3_.

Another solution is to measure the OiW concentration in real-time by incorporating a trailing moving average window. The size of the moving average window is highly based on the plant’s dynamics. Based on the results in Figure 16, Figure 17, Figure 18 and Figure 19, both microscopes are able to measure the the OiW concentration and thus they have the potential to track the transient behavior when it occurs in an IWT process. The fluctuating real-time results obtained with both microscopes, using a trailing moving average window of 1 min, can be questioned to be sufficient to provide qualitative feedback to an operator. However, by extending the moving average window to 3 min, the real-time measurements of OiW concentration from both microscopes are naturally more consistent, as shown in Figure 21. Extending the moving average window is only valuable if the dynamic of the plant is not faster than the moving average window. The higher resolution of C_3_ caused the microscope to capture twice as many particles compared to C_2_, thus when using the moving average window, C_3_ yields a more stable measurement.

One important procedure that has not been addressed in this paper is the classification within both microscopy analyzers. Oil droplets in Experiment III have been classified and withdrawn from the rest of the observed particles. The classification process has not been addressed as the procedure are very different in each microscope. C_2_ entrust the operator in selecting the classification range of each measured variable. C_3_ uses machine learning by letting an operator train the classification process by manually defining the captured particles. The different results of PSDs shown in Figure 14 and Figure 15, could be a result of classifying differently. The results obtained by C_3_ are not representable around 13 µm if the PSD truly follows a log-normal distribution. Another consequence to the bimodal PSD obtained by C_3_ could be the installment right after the narrow view cell of C_2_, which may change the PSD of oil droplets by breakups or coalescence. Another statical consideration was addressed when outputting a PSD, as the PSD should be trustworthy if an operator should draw any conclusion based on the presented PSD. Masuda and Iinoya [21] presented an estimation for predicting the required number of particles to be counted for a certain accuracy. In this paper, δ of the PSDs shown in Figure 14 and Figure 15 were calculated based on the number of particles obtained within 30 min at two different OiW concentrations (55ppm and 400ppm ) for both microscopes. Based on the results in Table 7, a δ<5% was observed within a 95% confidence interval of all the presentable PSDs. If the δ<5% is not enough more particles must be counted, i.e., at δ=1% roughly 100,000–130,000 for C_3_ and 90,000–100,000 for C_2_ must be counted based on the measured OiW concentrations, respectively. Seen from another point of view, if a PSD update every 30 min is too slow, the allowed δ in the presented PSD must be defined to determine the minimum number of particles required in order to achieve a defined level of accuracy.

## 5. Conclusions

The paper presented an evaluation of two different online microscopy analyzers that utilize a high-resolution video camera for capturing images of the particles passing their view cell. The three main calibration parameters: threshold, edge strength/focus rejection, and DoF, were validated and discussed in order to address the difficulty in determining which particles are considered to be in focus. The main contribution of this paper includes experiments of how the calibration affects the microscopy outputs, which is used to minimize the uncertainty by selecting calibration parameters. The procedure for selecting the three different calibration parameters must be well documented by the manufacturer to increase reproducibility when installed in an offshore oil and gas process. It was discussed whether the manufacturer could accommodate to reduce the use of perception when calibrating the instrument by integrating auto-calibration or human-computer interaction to minimize the uncertainties related to calibrating the microscope. Both microscopes were able to discriminate the particle size distribution of three known particle sizes with high precision, indicating a successful calibration procedure for accurately measuring the particle sizes.

A fluorescence-based monitor was installed as a benchmark to evaluate both online microscopes’ performance to measure oil-in-water (OiW) concentrations, both for steady-state measurements and real-time purposes. The fluorescence-based monitor was selected due to previous work by Hansen et al. [6] showing promising results for measuring OiW concentrations. Both measurements from the microscopy analyzers were reasonable compared to the known OiW concentration and the measured concentration from the fluorescence-based monitor. Real-time measurements from both microscopy analyzers were achievable by post-processing the data captured by the microscopes and applying a trailing moving average window of 1 min. Although selecting the size of the moving average window should be based on the plant’s dynamics.

Lastly, a statical consideration was addressed to determine the minimum number of particles required in order to achieve a defined level of accuracy within a defined confidence interval when outputting the particle size distribution to an operator.

## Figures and Tables

**Table 1:** Execution specifications of both microscopes.

	Jorin ViPA	Canty InFlow
Pixel scale	0.375 µm/pixel	0.513 µm/pixel
Pixel dimensions	1292×964 pixels	1920×1200 pixels
Field of view	484.50×361.50 µm	984.96×615.60 µm
Depth of field *	116.50 µm	93.03 µm
Frame rate	∼30 fps	∼30 fps
Inlet/outlet ports	1/4${}^{\prime \prime }$	1/2${}^{\prime \prime }$
Flow velocity *	0.03–2.1 m/s	0.25–2.74 m/s
Flow rate *	0.05–4 L/min	1.9–20.8 L/min

* Information provided by the manufacturers

**Table 2:** A list of each wanted OiW concentration by addition of specific amount of oil. $V_{i}$ represent the total amount of oil added, where $V_{i}-V_{i-1}$ represents the amount that should be added to reach the next OiW concentration.

Wanted [ppm]	$V_{i}$ [mL]	$V_{i}-V_{i-1}$ [mL]
0	0	0
55	9.00	9.00
100	16.36	7.36
150	24.54	8.18
200	32.72	8.18
250	40.91	8.18
400	65.46	24.56

**Table 3:** Associated results to Figure 6, based on different ESVs.

Edge Strength	Size	Aspect Ratio	Shape Factor
0	6.53	0.69	0.36
1	16.86	0.92	0.86
2	15.59	0.92	0.87
3	17.18	0.87	0.89
4	17.27	0.96	0.97
5	19.82	0.99	0.94
6	19.56	0.97	0.96
7	19.43	0.99	0.97
8	20.50	0.96	0.93
9	19.56	0.97	0.96
10	19.97	0.97	0.98

**Table 4:** Associated results to Figure 7.

Edge Strength	Size	Aspect Ratio	Shape Factor
≤1	19.70	0.94	0.80
2	1.81	0.71	0.96
≥3	−	−	−

**Table 5:** Associated results to Figure 10.

	Known μ(σ) [µm]	$\mu_{\mathrm{estimat}.}$ [µm]	$\sigma_{\mathrm{estimat}.}$ [µm]
	9.8(0.3)	9.5	0.6
C_3_	20.1(0.4)	20.0	0.6
	40.3(0.9)	40.9	0.5
	9.8(0.3)	9.6	0.6
C_2_	20.1(0.4)	20.3	1.0
	40.3(0.9)	41.4	0.6

**Table 6:** Associated results to Figure 13.

Known Conc.		55 ppm	100 ppm	150 ppm	200 ppm	250 ppm	400 ppm
	minimum:	56.2	94.7	146.1	193.9	237.6	379.0
C_1_	μ:	60.9	100.1	151.8	200.7	244.6	390.0
	maximum:	67.7	105.4	159.5	207.7	255.8	398.4
	minimum:	31.6	71.4	118.0	153.5	183.7	331.9
C_2_	μ:	49.2	106.1	168.9	215.7	263.1	393.6
	maximum:	75.1	177.8	230.7	309.3	373.4	478.0
	minimum:	44.9	84.6	136.9	172.4	231.2	362.6
C_3_	μ:	55.1	104.0	162.4	210.0	259.3	410.6
	maximum:	72.3	136.1	189.2	239.6	289.4	451.7

**Table 7:** Associated results to Figure 14 and Figure 15, calculating the , related to each PSD of each OiW concentration measured with C_2_ and C_3_.

Known OiW Conc.		55 ppm	400 ppm
C_2_	$\mu_{0}$:	11.7	13.6
	$\sigma_{0}$:	7.3	8.9
	$X_{N}$:	6733	27154
	δ	3.5%	1.9%
C_3_	$\mu_{0}$:	24.3	29.0
	$\sigma_{0}$:	16.2	21.4
	$X_{N}$:	11068	45963
	δ:	3.0%	1.6%

## Data Availability

The data presented in this study are available on request from the corresponding author.

## Abbreviations

The following abbreviations are used in this manuscript:

$C_{1}$	Turner TD-4100XDC
$C_{2}$	Jorin ViPA B HiFlo
$C_{3}$	Canty InFlow VD4912-960
CP	Centrifugal pump
DoF	Depth of field
ESV	Edge strength
fps	frames per second
FRV	Focus rejection value
HCI	Human-computer interaction
IW	Injection water
IWT	Injection water treatment
TSS	Ttotal suspended solids
OiW	Oil-in-water
$P_{x}$	Pressure transmitters
PDF	Probability density function
PSD	Particle size distribution
PW	Produced water
PWRI	Produced water re-injection
$Q_{x}$	Flowmeters
THV	Threshold value
$V_{x}$	Pneumatic control valves
